# Comparison of measures of comorbidity for predicting disability 12-months post-injury

**DOI:** 10.1186/1472-6963-13-30

**Published:** 2013-01-26

**Authors:** Belinda J Gabbe, James E Harrison, Ronan A Lyons, Elton R Edwards, Peter A Cameron

**Affiliations:** 1 Department of Epidemiology and Preventive Medicine, Monash University, The Alfred Centre, 99 Commercial Rd, Melbourne, Victoria, 3004, Australia; 2 National Trauma Research Institute, The Alfred Hospital, Melbourne, Australia; 3 Research Centre for Injury Studies, Flinders University, Adelaide, Australia; 4 College of Medicine, Swansea University, Swansea, United Kingdom; 5 Department of Orthopaedic Surgery, The Alfred, Melbourne, Australia; 6 Emergency and Trauma Centre, The Alfred Hospital, Melbourne, Australia

**Keywords:** Orthopaedic injury, Comorbidity, Disability outcomes, Prediction

## Abstract

**Background:**

Understanding the factors that impact on disability is necessary to inform trauma care and enable adequate risk adjustment for benchmarking and monitoring. A key consideration is how to adjust for pre-existing conditions when assessing injury outcomes, and whether the inclusion of comorbidity is needed in addition to adjustment for age. This study compared different approaches to modelling the impact of comorbidity, collected as part of the routine hospital episode data, on disability outcomes following orthopaedic injury.

**Methods:**

12-month Glasgow Outcome Scale – Extended (GOS-E) outcomes for 13,519 survivors to discharge were drawn from the Victorian Orthopaedic Trauma Outcomes Registry, a prospective cohort study of admitted orthopaedic injury patients. ICD-10-AM comorbidity codes were mapped to four comorbidity indices. Cases with a GOS-E score of 7–8 were considered “recovered”. A split dataset approach was used with cases randomly assigned to development or test datasets. Logistic regression models were fitted with “recovery” as the outcome and the performance of the models based on each comorbidity index (adjusted for injury and age) measured using calibration (Hosmer-Lemshow (H-L) statistics and calibration curves) and discrimination (Area under the Receiver Operating Characteristic (AUC)) statistics.

**Results:**

All comorbidity indices improved model fit over models with age and injuries sustained alone. None of the models demonstrated acceptable model calibration (H-L statistic p < 0.05 for all models). There was little difference between the discrimination of the indices for predicting recovery: Charlson Comorbidity Index (AUC 0.70, 95% CI: 0.68, 0.71); number of ICD-10 chapters represented (AUC 0.70, 95% CI: 0.69, 0.72); number of six frequent chronic conditions represented (AUC 0.70, 95% CI: 0.69, 0.71); and the Functional Comorbidity Index (AUC 0.69, 95% CI: 0.68, 0.71).

**Conclusions:**

The presence of ICD-10 recorded comorbid conditions is an important predictor of long term functional outcome following orthopaedic injury and adjustment for comorbidity is indicated when assessing risk-adjusted functional outcomes over time or across jurisdictions.

## Background

Measuring disability following injury is important. Understanding the factors that impact on disability is necessary to inform trauma care, and enable adequate risk-adjustment for benchmarking and monitoring. The presence of comorbidities has been identified as an important predictor of mortality following injury [1-3]. Others have found that age is a more important predictor, and comorbidity offers little prediction benefit compared to models using age alone [4-6]. The impact of comorbidity on the prediction of disability outcomes has not been well explored. Untangling the effect of comorbidity has been identified as an important challenge for measuring injury-related disability [7,8].

De Groot *et al.*, identified numerous methods for measuring comorbidity, and highlighted many issues with the development and validation of comorbidity indices [9]. Of note were the lack of a consistent definition of comorbidity, variation in populations used to develop comorbidity measures, limited validation undertaken, and few measures developed with disability in mind [9]. Several studies have reported associations between comorbidity and disability [7,10-16] but none have directly compared indices.

This study compared different approaches to modelling the impact of comorbidity, collected as part of the routine hospital discharge data, on 12-month disability outcomes in an orthopaedic injury population to inform the development of risk adjustment models for disability outcomes. A key additional aim was to establish whether the inclusion of comorbidity provides additional important predictive value in addition to adjustment for age alone.

## Methods

### Data

The Victorian Orthopaedic Trauma Outcomes Registry (VOTOR) monitors the management and outcomes following admission to hospital for adults with acute orthopaedic injury [17]. This sentinel site registry collects data about all orthopaedic trauma admissions to four participating hospitals in the state of Victoria, Australia. The contributing hospitals include two adult major trauma services (Level 1 trauma centre equivalent), one large regional (rural) hospital, and one large metropolitan hospital, to ensure a wide representation of orthopaedic trauma patients in the registry. All eligible cases are included on the registry using an opt-off consent process [18]. The registry is approved by the Human Research Ethics Committee at each participating hospital and Monash University.

### Procedures

Cases included survivors to hospital discharge, admitted between March 2007 and July 2010. Demographic, injury event, International Classification of Diseases 10th revision Australian Modification (ICD-10-AM) diagnosis codes (up to 40 per admission), and 12-month functional outcomes data were extracted for analysis.

ICD-10-AM orthopaedic injury diagnosis codes were mapped to 10 orthopaedic injury groups. Indicator variables were generated to represent important non-orthopaedic injuries, including variables for intracranial injury and/or skull fracture, multiple rib fractures, intra-abdominal or intra-thoracic organ injury, and burns.

### Comorbidity measures

ICD-10-AM diagnosis codes are the source of comorbidities for VOTOR. In Victoria, each code contains a prefix with “P” representing principal diagnosis requiring treatment during the stay, “A” representing additional diagnoses and “C” representing in-hospital complications. Australian Coding Standards specify that most conditions should be coded as additional diagnoses if they affect treatment, require investigation, or use resources during the admission. However, the standards require coding of certain conditions whenever they are present; some communicable diseases (e.g. HIV, viral hepatitis), diabetes and pregnancy [19].

For the purposes of this study, the following were excluded from mapping to comorbidity measures:

i. All in-hospital complications (“C” prefix codes);

ii. Chapter XIX (Injury, poisoning and certain other consequences of external causes) with a “P” prefix (indicating an injury principal diagnosis);

iii. All Chapter XVIII (Symptoms, signs and abnormal clinical and laboratory findings, not elsewhere classified) as these are not comorbidities;

iv. Chapters XX (External causes of morbidity and mortality) to XXII (Codes for special purposes) as these are not comorbidities.

Remaining “P” and “A” prefixed codes were checked by the authors (PAC, RAL, BJG) to remove erroneously coded complications. These included acute post-haemmorhagic anaemia, acute subendocardial myocardial infarction, acute renal failure, acute respiratory infections, fever and post-traumatic amnesia. Codes remaining were assumed to represent comorbidities.

Four methods for classifying comorbidity were used and the ICD-10-AM codes mapped to the four indices. These were: (i) Charlson Comorbidity Index (CCI); (ii) Functional Comorbidity Index (FCI); (iii) categorisation by ICD-10 chapter; and (iv) the six conditions described by Haagsma *et al.*[7].

#### Charlson comorbidity index (CCI)

The 19 Charlson conditions were mapped to the CCI from the ICD-10-AM codes (Table 1), resulting in a weight of 1, 2, 3 or 6 in accordance with Charlson’s recommendations, and zero if no CCI codes were recorded [5]. The CCI is used widely for outcome adjustment in the injury literature [10,11,14].

**Table 1:** ICD-10 to comorbidity indices map

**Comorbidity Measure**		**ICD-10 codes**
Functional Comorbidity Index	Arthritis	M05.0-M05.9, M06.0-M06.9, M08.0-M08.9, M13.0-M13.9, M15.0-M19.9, M47.0-M47.9, M48.0, M48.9
	Osteoporosis	M80.0-M80.9, M81.0-M81.9, M82.0-M82.9, M83.0-M83.9
	Asthma	J45.0-J45.9
	COPD/ARDS	J43.0-J44.9
	Angina	I20.0-I20.9
	CHF or Heart disease	I05.0-I07.9, I10.0-I11.9, I13.0-I15.9, I24.0-I24.9, I25.0-I25.1, I25.3-I25.9, I27.0-I27.9, I31.0-I31.9, I34.0-I35.9, I42.0-I42.9, I44.0-I46.9, I47.0-I51.9
	Heart Attack	I21.0-I21.9, I22.0-I22.9, I25.2
	Neurological disease	G00.0-G99.9
	Stroke or TIA	I60.0-I64.9, G45.0-G45.9
	Diabetes	E10.0-E14.9
	PVD	I73.9, I70.2
	Upper GI disease	K20.0-K22.9, K25.0-K31.9
	Depression	F32.0-F32.9, F33.0-F33.9
	Anxiety or panic disorders	F40.0-F40.9, F41.0-F41.9
	Visual impairment	H53.0-H54.9
	Hearing impairment	H90.0-H91.9
	Degenerative disc disease	M50.0-M51.9
	Obesity	E66.0-E66.9
Haagsma et al. (2011)	Chronic non-specific lung disease	J45.0-J45.9, J43.0-J44.9
	Heart disease	I05.0-I07.9, I10.0-I11.9, I13.0-I15.9, I24.0-I24.9, I25.0-I25.1, I25.3-I25.9, I27.0-I27.9, I31.0-I31.9, I34.0-I35.9, I42.0-I42.9, I44.0-I46.9, I47.0-I51.9, I20.0-I20.9, I21.0-I21.9, I22.0-I22.9, I25.2
	Diabetes	E10.0-E14.9
	Backache	M48.0, M51.0, M51.1, M51.3-M51.9, M54.3-M54.6, M54.9
	OA	M13.0-M13.9, M15.0-M19.9, M47.0-M47.9, M48.0, M48.9
	RA	M05.0-M05.9, M06.0-M06.9, M08.0-M08.9
	Other disease or injury	A00.0-B99.9, C00.0-C97.9, D00.0-D48.9, D50.0-D58.9, E00.0-E07.0, E15.0-E90.9, F00.0-F99.9, G00.0-G99.9, H00.0-H59.9, H60.0-H95.9, I00.0-I99.9, J00.0-J39.9, J40.0-J42.9, J46.0-J99.9, K00.0-K93.9, L00.0-L99.9, M00.0-M03.9, M07.0-M07.9, M09.0-M12.9, M14.0-M14.9, M20.0-M46.9, M48.1-M48.8, M49.0-M99.9, N00.0-N99.9, O00.0-O99.9, P00.0-P96.9, Q00.0-Q99.9, R00.0-R99.9, S00.0-S99.9, T00.0-T98.9
Charlson Comorbidity Index	Myocardial infarction	I21-I24, I25.2, I25.8
	Congestive heart failure	I09, I11, I48, I49.0, I49.8, I50.0, I50.1, I50.1 + J81, I50.9, I51.5, I97.1
	Peripheral vascular disease	I70, I71.1-I71.6, I71.8-I71.9, 172, I73, I74, I77
	Dementia	F00-F04, F05.1, F10
	Cerebrovascular disease	I60-I63, I65-I68, G45
	Chronic pulmonary disease	J41-J47
	Connective tissue disease	M05-M06, M08-M09, M12-M13, M30-M36
	Ulcer disease	K25-K28
	Mild liver disease	K70.0-K70.3, K70.9, K73, K74, K75.2-K75.9, K76.0-K76.5, K76.8-K76.9, K77
	Diabetes	E10, E10.1, E10.5-E10.9, E11, E11.1, E11.5-E11.9, E13, E13.1, E13.5-E13.9, E14, E14.1, E14.5-E14.9
	Hemiplegia	G81.0-G81.1, G81.9, I63, I66-I67
	Moderate or severe renal disease	I12-I13, N00-N05, N17-N19
	Diabetes with end-organ damage	E10.2-E10.4, E11.2-E11.4, E13.2-E13.4, E14.2-E14.4
	Any tumour	D00-D48
	Leukaemia	C91-C95
	Lymphoma	C81-C85
	Moderate or severe liver disease	K70.4, K71.1, K71.7, K72, K75.0-K75.1, K76.6-K76.7
	Metastatic solid tumour	C00-C26, C30-C34, C37-C41, C43-C58, C60-C80, C88, C90, C96
	AIDS	B20-B24

#### Functional comorbidity index (FCI)

The FCI was developed as a comorbidity index with physical function as the outcome of interest, using an 18-item (comorbidities) self-administered questionnaire where the FCI score is the sum of the number of conditions reported for the person (0–18) [20]. ICD-10-AM codes were mapped to the indicator variables (yes/no) for each FCI condition (Table 1) and FCI scores calculated as the sum of the conditions represented.

#### ICD-10 chapters

Indicator variables were generated for the presence or absence of conditions in Chapters I to XVII, and Chapter XIX. A variable indicating the number of chapters represented was generated, an approach used by Cameron *et al.*[10].

#### Six frequent chronic conditions described by Haagsma et al. (2011)

Haagsma *et al.* investigated the impact of comorbidity on disability weight estimates in a sample of 2,295 injured patients in the Netherlands [7]. The six most common self-reported chronic diseases were: (i) chronic non-specific lung disease; (ii) heart disease; (iii) diabetes; (iv) backache; (v) osteoarthritis; and (vi) rheumatoid arthritis. All other comorbidities are considered as “other”. Indicator variables for these conditions were mapped from the ICD-10 codes (Table 1).

The key differences between the comorbidity indices used relate to the number of conditions represented. The ICD chapter approach maps all available ICD-10 comorbidity codes into 18 chapter-related groups, but does not specifically identify individual conditions. For example, diabetes is included in the “Endocrine, nutritional and metabolic disorders” chapter. The six frequent chronic conditions described by Haagsma et al. [7] also uses all available ICD-10 comorbidity codes but only six specific diagnoses are included, with all remaining comorbidities grouped together in an “other” category. The FCI includes only 18 conditions, with patients’ ICD-10 coded comorbidities not included in this list considered as having no comorbidities. The ICD-10 chapter, six frequent chronic conditions and FCI do not weight the severity of comorbidities. The CCI includes 19 conditions, but weights the severity of these conditions. For example, the presence of diabetes is given either a weighting of 1 or 2 depending on whether there is end-organ disease, and mild liver disease is differentiated from moderate/severe liver disease by CCI weightings.

The relationship between the conditions included in each comorbidity measure is complex. The ICD-10 chapter approach includes *all* conditions specified by the six frequent chronic condition, FCI and CCI comorbidity measures. All remaining measures include specific categories for heart disease, chronic pulmonary disease, and diabetes, although how this is represented varies (Table 1). For example, the six frequent common conditions approach bundles all diagnoses related to heart disease into a single category, while the FCI separates heart disease into three categories; angina, congestive heart failure (CHF)/heart disease, and heart attack. The CCI uses two categories for heart disease; myocardial infarction, and CHF. Arthritis is common to the six frequent chronic conditions and FCI approach, although the six frequent chronic conditions approach separates rheumatoid arthritis and osteoarthritis into individual categories. Similarly, the six frequent common conditions approach includes a category for “backache” while the FCI includes only a subset of relevant ICD-10 codes in its degenerative disc disease category (Table 1). Both the FCI and CCI include a specific category for peripheral vascular disease. Gastrointestinal and neurological disease are included in both the FCI and CCI but the specificity of categories differs between the two measures (Table 1). Only the FCI includes osteoporosis, obesity, depression, anxiety disorders, vision impairment and hearing impairment as individual categories, while only the CCI includes connective tissue disease, dementia, renal disease, metastatic disease, AIDS, leukemia, lymphoma, and other tumours as specific categories. Overall, the common groups of conditions represented in *all* comorbidity measures are diabetes, heart disease, and chronic pulmonary disease.

### Outcome measures

Survivors to discharge in VOTOR are followed-up by standardised telephone interview at 12-months post-injury to collect disability outcomes [18]. The outcome of interest for this study was the Glasgow Outcome Scale – Extended (GOS-E) which rates a person’s function on an 8-level scale from 1 (death) to 8 (upper good recovery) [21] .The GOS-E is recommended for measurement of trauma patient outcomes [22,23], has demonstrated high levels of responsiveness to change in patients without head injury [24], and considers the patient’s pre-injury function in the scoring process [21]. The GOS-E was dichotomised with a score <7 representing ongoing disability and 7–8 “recovery”.

### Data analysis

A split dataset approach was used with the dataset randomly divided into two equal parts [25]. Models were developed using the “training” dataset and internally validated using the “test” dataset. Means and standard deviations (SD), or medians and interquartile range (IQR), were used to summarise continuous variables. Categorical variables were summarised using counts and percentages. Logistic regression models were fitted with “recovery” as the outcome. Model performance was measured using calibration and discrimination statistics. Age was categorised into eight groups, as age in its continuous form was not linearly related to the log odds of recovery.

Calibration measures how well the models predict over the entire range and was assessed using the Hosmer-Lemeshow (H–L) statistic and calibration curves. A higher H-L statistic and significant p-value correspond to poorer calibration [26]. Calibration curves plot the observed proportion of events against the predicted probabilities of events, with perfect agreement between observed and predicted probabilities forming a 45° line (“line of best fit”) [25].

The area under the receiver operating characteristic (ROC) curve (AUC) measures the capacity of the model to discriminate between those who do and do not experience the outcome of interest [25]. ROC plots sensitivity against 1-specificity over the range of probabilities. Discrimination is generally classified as Acceptable (AUC 0.7 - <0.8), Excellent (AUC 0.8 - <0.9) and Outstanding (AUC ≥0.9) [26].

In the training dataset, a model was fitted with injury variables and age as independent variables, and “recovery” as the dependent variable. Each measure of comorbidity was added and the models were compared using likelihood ratio (LR) tests, AUC (95% CI), and the H-L statistic. The following comorbidity adjustment methods were used.

i. CCI weight categorised as 0, 1, ≥ 2

ii. Number of ICD-10 chapters represented categorised as 0, 1, ≥ 2

iii. FCI score categorised as 0, 1, ≥ 2

iv. Number of the six frequent chronic conditions described by Haagsma et al. [7] represented categorised as 0, 1, ≥ 2

v. All indicator variables for ICD-10 chapters included

vi. All FCI condition indicator variables included

vii. All of the six frequent chronic condition indicator variables included.

The number of conditions/weighted index was not linearly related to the log odds of recovery, requiring categorisation. The individual conditions of the CCI were not modeled as the weighted index is the most commonly used form of the index. Models were then fitted to the test dataset and fit assessed using the AUC, H-L statistics, and calibration curves. Data were complete for all data items included in the models, ensuring that comparison between models was based on the same cases. All analyses were performed using Stata Version 11.0 (Stata Corp, College Station, Texas). A p-value <0.05 was considered significant.

## Results

### Overview of the dataset

There were 15,471 survivors to discharge, and 13,519 (87.4%) had a valid GOSE-E at 12-months. Cases lost to follow-up at 12-months included a higher proportion of patients less than 45 years of age, male, and injured in motor vehicle crashes (Table 2). The overall distribution of orthopaedic injuries sustained was comparable between the groups, but the proportion without documented comorbidities able to be coded to the indices of interest was higher in the cases lost to follow-up (Table 2).

**Table 2:** Comparison of orthopaedic trauma patients followed-up at 12-months and patients lost to follow-up

**Population characteristic**		**Followed-up (n = 13,519)**	**Lost to follow-up (n = 1,952)**
Age group	15-24 years	1704 (12.6)	356 (18.2)
	25-34 years	1672 (12.4)	404 (20.7)
	35-44 years	1694 (12.5)	301 (15.4)
	45-54 years	1655 (12.2)	220 (11.3)
	55-64 years	1577 (11.7)	199 (10.2)
	65-74 years	1480 (10.9)	157 (8.0)
	75-84 years	2071 (15.3)	200 (10.3)
	85+ years	1666 (12.3)	115 (5.9)
Gender	Male	7361 (54.5)	1204 (61.7)
	Female	6158 (45.5)	748 (38.3)
Mechanism of injury	Low fall (<1 m)	5390 (39.9)	613 (31.4)
	Motor vehicle	1869 (13.8)	339 (17.4)
	High fall	1816 (13.4)	254 (13.0)
	Motorcycle	1349 (10.0)	208 (10.7)
	Pedestrian	579 (4.3)	122 (6.3)
	Pedal cyclist	616 (4.6)	71 (3.6)
	Collision with object or person	505 (3.7)	120 (6.1)
	Other	1395 (10.3)	225 (11.5)
Injury group	Isolated lower extremity fracture	5271 (39.0)	701 (35.9)
	Isolated upper extremity fracture	2809 (20.8)	478 (24.5)
	Spinal fractures only	1741 (12.9)	254 (13.0)
	Multiple lower extremity fractures	972 (7.2)	123 (6.3)
	Upper and lower extremity fractures	620 (4.6)	87 (4.5)
	Soft tissue injury	540 (4.0)	94 (4.8)
	Spine and lower extremity fractures	465 (3.4)	64 (3.3)
	Multiple upper extremity fractures	440 (3.3)	69 (3.5)
	Spine and upper extremity fracture	431 (3.2)	50 (2.6)
	Spine, upper and lower extremity fractures	230 (1.7)	32 (1.6)
CCI^a^ weight	0	9801 (72.5)	1489 (76.3)
	1	2681 (19.8)	365 (18.7)
	≥2	1037 (7.7)	98 (5.0)
FCI^b^ score	0	10859 (80.3)	1675 (85.8)
	1	1951 (14.4)	212 (10.9)
	≥2	709 (5.2)	65 (3.3)
ICD-10^c^ chapters	0	3515 (26.0)	554 (28.4)
	1	2949 (21.8)	441 (22.6)
	≥2	7055 (52.2)	957 (49.0)
Haagsma conditions [7]	0	8336 (61.7)	1259 (64.5)
	1	3979 (29.4)	594 (30.4)
	≥2	1204 (8.9)	99 (5.1)

^a^CCI, Charlson Comorbidity Index; ^b^FCI, Functional Comorbidity Index; ^c^ICD-10, International Classification of Diseases, 10th Revision.

Most cases followed-up at 12-months post-injury had no comorbidity recorded in the ICD-10-AM diagnoses (Table 3). Mental and behavioural disorders (14%), diseases of the circulatory system (10%), and endocrine, nutritional and metabolic disorders (7%) were most prevalent when using the ICD-10 chapters to classify comorbidity. Using the FCI, diabetes (6%), heart disease (5%) and neurological disease (5%) were most prevalent, while “other” conditions (34%) were most common using the six frequent chronic conditions described by Haagsma *et al.* (Table 3).

**Table 3:** Distribution of comorbid conditions in the dataset (n = 13,519)

**Comorbidity measure**			
**ICD-10**^**a**^**Chapters**	**n (%)**	**FCI**^**b**^**conditions**	**n (%)**
I – Infectious and parasitic diseases	470 (3.5)	Arthritis	145 (1.1)
II – Neoplasms	178 (1.3)	Osteoporosis	296 (2.2)
III – Diseases of blood and blood-forming organs	723 (5.4)	Asthma	25 (0.2)
IV – Endocrine, nutritional, metabolic disorders	900 (6.7)	COPD/ARDS^c^	225 (1.7)
V – Mental and behaviour disorders	1950 (14.4)	Angina	23 (0.2)
VI – Diseases of the nervous system	783 (5.8)	Congestive heart failure/Heart disease	726 (5.4)
VII- Diseases of the eye and adnexa	288 (2.1)	Heart attack	73 (0.5)
VIII – Diseases of the ear and mastoid process	130 (1.0)	Neurological disease	656 (4.9)
IX – Diseases of the circulatory system	1349 (10.0)	Stroke or Transient Ischaemic Attack	37 (0.3)
X – Disease of the respiratory system	424 (3.1)	Diabetes	815 (6.0)
XI – Diseases of the digestive system	347 (2.6)	Peripheral vascular disease	56 (0.4)
XII – Diseases of the skin, subcutaneous tissue	332 (2.5)	Upper gastrointestinal disease	62 (0.5)
XIII – Diseases of the musculoskeletal system	806 (6.0)	Depression	22 (0.2)
XIV – Diseases of the genitourinary system	543 (4.0)	Anxiety/panic disorders	80 (0.6)
XV – Pregnancy, childbirth and the puerperium	3 (0.02)	Visual impairment	101 (0.8)
XVI – Conditions originating in perinatal period	1 (0.01)	Hearing impairment	93 (0.7)
XVII – Congenital malformations	52 (0.4)	Degenerative disc disease	21 (0.2)
XIX – Injury, poisoning, etc.	83 (0.6)	Obesity	135 (1.0)
**Six frequent chronic conditions**[7]	**n (%)**	**Charlson Comorbidity Index**	**n (%)**
Chronic lung disease	250 (1.9)	Myocardial infarction	238 (1.8)
Heart disease	782 (5.8)	Congestive heart failure	378 (2.8)
Diabetes	815 (6.0)	Peripheral vascular disease	19 (0.1)
Backache	49 (0.4)	Dementia	1898 (14.0)
Osteoarthritis	129 (1.0)	Cerebrovascular disease	46 (0.3)
Rheumatoid arthritis	17 (0.1)	Chronic pulmonary disease	277 (2.0)
Other	4555 (33.7)	Connective tissue disease	37 (0.3)
		Ulcer disease	19 (0.1)
		Mild liver disease	98 (0.7)
		Diabetes	524 (3.9)
		Hemiplegia	155 (1.1)
		Moderate or severe renal disease	547 (4.0)
		Diabetes with end-organ damage	379 (2.8)
		Any tumour	65 (0.5)
		Leukaemia	13 (0.1)
		Lymphoma	2 (0.01)
		Moderate or severe liver disease	30 (0.2)
		Metastatic solid tumour	105 (0.8)
		AIDS	16 (0.1)

^a^ICD-10, International Classification of Diseases, 10th Revision; ^b^FCI, Functional Comorbidity Index; ^c^COPD, Chronic Obstructive Pulmonary Disease, ARDS, Adult Respiratory Distress Syndrome.

The random split resulted in 6,798 cases in the training dataset and 6,792 cases in the test dataset. Cases in the two datasets were comparable. The percentage of cases who had “recovered” by 12-months post-injury was 42% in both datasets.

### Contribution of age and comorbid conditions to prediction of 12-month disability

#### Training dataset

Adding age resulted in improved model fit over adjustment for injuries alone (Table 4). The addition of comorbid status, irrespective of method of comorbid measurement, improved model fit further (Table 4). All comorbidity adjustment methods resulted in acceptable calibration (as tested using the H-L statistic), but use of the ICD-10 chapters and the six frequent chronic conditions approaches demonstrated higher AUC than adjustment using the FCI or CCI (Table 4). Adjustment for the number of comorbid conditions compared to adjusting for the presence or absence of each condition/chapter did not result in improved discrimination for the ICD-10 chapters (Χ^2^_1_ = 0.11, p = 0.743), FCI (Χ^2^_1_ = 0.37, p = 0.544) or the six frequent chronic conditions (Χ^2^_1_ = 1.21, p = 0.271). The AUC was higher for the number of ICD-10 chapters represented when compared to the number of the six frequent chronic conditions represented (Χ^2^_1_ = 8.75, p = 0.003). However, the overall range of the AUC for the models adjusting for comorbidity ranged from 0.716 to 0.729 (Table 4).

**Table 4:** Discrimination and calibration of models in training dataset (n = 6798)

**Model**	**Comorbidity measure**	**Area under curve (95% CI)**	**H-L statistic (p-value)**	**LR test (p-value)**
Injury group	None	0.631 (0.618, 0.644)	0.67 (0.954)	-
Injury group and age group	None	0.704 (0.692, 0.716)	4.04 (0.854)	507.40 (<0.0001)
Injury group, age group and comorbidity	Number of ICD-10 chapters	0.728 (0.716, 0.740)	11.90 (0.156)	228.23 (<0.0001)*
Injury group, age group and comorbidity	ICD-10 chapters	0.729 (0.717, 0.741)	8.51 (0.386)	251.38 (<0.0001)*
Injury group, age group and comorbidity	Number of Haagsma conditions	0.724 (0.712, 0.736)	7.51 (0.482)	182.06 (<0.0001)*
Injury group, age group and comorbidity	Haagsma conditions	0.725 (0.713, 0.737)	7.05 (0.531)	194.03 (<0.0001)*
Injury group, age group and comorbidity	CCI weight category	0.720 (0.708, 0.732)	1.30 (0.996)	159.35 (<0.0001)*
Injury group, age group and comorbidity	FCI score	0.716 (0.704, 0.728)	7.10 (0.526)	102.39 (<0.0001)*
Injury group, age group and comorbidity	FCI conditions	0.716 (0.704, 0.728)	8.05 (0.429)	-

*compared to model including age group and injury group.

#### Test dataset

**Figure 1:**
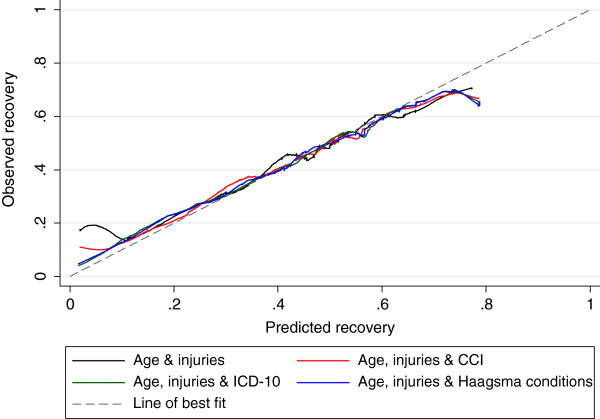
**The figure is a plot the predicted versus the observed recovery in the test dataset.** The 45° line represents perfect fit of the model.

Fitting the models to the test dataset resulted in a similar pattern of results but poorer model fit with none of the models demonstrating acceptable calibration and lower AUC than the training dataset models (Table 5). The AUC ranged from 0.691 to 0.704 with the number of ICD-10 chapters represented demonstrating the highest discrimination (Table 5). Despite poor calibration of models as measured using the H-L statistic, calibration curves tracked close to the line of best fit with the ICD-10 and six frequent common condition curves showing better calibration at lower prediction percentiles and all models over-estimating recovery at higher prediction percentiles (Figure 1).

**Table 5:** Discrimination and calibration of models in test dataset (n = 6721)

**Model**	**Comorbidity measure**	**Area under curve (95% CI)**	**H-L statistic (p-value)**
Injury group	None	0.600 (0.587, 0.613)	35.52 (<0.0001)
Injury group and age group	None	0.678 (0.665, 0.690)	25.64 (0.001)
Injury group, age group and comorbidity	Number of ICD-10 chapters	0.704 (0.692, 0.717)	19.12 (0.014)
Injury group, age group and comorbidity	ICD-10 chapters	0.703 (0.691, 0.716)	22.61 (0.004)
Injury group, age group and comorbidity	Number of Haagsma conditions	0.701 (0.689, 0.713)	20.31 (0.009)
Injury group, age group and comorbidity	Haagsma conditions	0.701 (0.688, 0.713)	18.47 (0.018)
Injury group, age group and comorbidity	CCI weight category	0.696 (0.683, 0.708)	19.78 (0.011)
Injury group, age group and comorbidity	FCI score	0.694 (0.681, 0.706)	16.71 (0.033)
Injury group, age group and comorbidity	FCI conditions	0.691 (0.678, 0.703)	18.84 (0.016)

## Discussion

The potential for comorbidity to impact on the long term disability experienced by injury survivors is clear but how best to adjust for comorbidity has not been well explored. This study of 13,519 injury survivors found that comorbidity, mapped to commonly used indices from routinely collected ICD-10 diagnosis codes, is an important predictor of functional recovery, providing additional predictive value over adjustment for age alone.

The findings confirm that comorbidity impairs patient recovery after injury. However, measurement of comorbidity remains a challenge. A lack of defined criteria for what constitutes a comorbidity, and absence of an established gold standard for measuring comorbidity [3,9], have led to a variety of methods and indices being used in injury research. Routinely collected discharge data, patient self-report and medical record review are common sources of comorbidity information [27,28]. Each source has strengths and weaknesses with self-report criticised due to the potential for recall bias, difficulty in data collection with cognitive deficits, and prohibitive costs in large studies [28]. Medical record review is considered the most comprehensive method, with documented conditions corresponding well to established comorbidity indices, but is resource intensive [28]. In the current study, routinely collected discharge data were used to map ICD-10 diagnoses to comorbidity indices. Routinely collected discharge data have been described as “inevitably imperfect” [28] due to the coding of only a subset of recorded conditions and a focus on coding for maximising reimbursement in many settings. However, routinely collected discharge data provide a less resource intensive method for capturing comorbidities and have been shown to agree with medical record review for key conditions such as diabetes, cancer, chronic lung disease and alcohol abuse [29]. Studies directly comparing various data sources in trauma are absent, but Fan *et al.*, in their study of >10,000 Veterans Affairs patients, found comparable prediction of health-related quality of life when comparing routinely collected discharge data with record review and self-report [27].

Our findings are consistent with previous studies that have found comorbidity based on patient or proxy self-report [7,12,13,15,16] and registry data, [11] to be an important predictor of longer term functional or health-related quality of life outcome following injury. In contrast to previous studies, comorbidity provided additional predictive value over adjustment only for age [3-5]. Adjustment for the specific conditions showed little improvement over adjustment based on the number of conditions represented for the ICD-10 chapters, FCI and the six frequent chronic conditions reported by Haagsma *et al.*, supporting methods of adjustment previously used [10,11,15,16].

Discrimination and calibration statistics of the various models revealed relatively little difference in model prediction of functional recovery, with ICD-10 chapter and the six frequent chronic condition-based models demonstrating slightly better performance than FCI and CCI models. A potential explanation for the improved performance of the ICD-10 and the six frequent chronic condition models is that they used all of the available ICD-10 codes, and therefore all of the comorbidity information available, while the FCI and CCI restricted the conditions included in the model to a specific subset. In data not shown, only diabetes, heart disease, stroke, neurological disease, PVD and visual impairment were significant predictors of recovery in the FCI models. For the six frequent chronic condition models, diabetes, heart disease and “other” conditions remained significant. The ICD-10 chapters related to ear, respiratory, skin, musculoskeletal, digestive, genitourinary and congenital conditions failed to reach significance in the model.

The prevalence of osteoarthritis, rheumatoid arthritis and obesity in the study population was 1%, 0.1% and 1% (Table 2). The reported prevalence of these diseases in the Australian population is 15%,[30] 2%,[31] and 19-22% [32], respectively. In contrast, the prevalence of heart disease in the study population was 6%, slightly higher than the reported Australian population prevalence for heart, stroke and vascular conditions of 4% [33]. The prevalence of diabetes in the study population was 6%, also slightly higher than the reported 4% national prevalence [34]. The differences in sample prevalence of diseases relative to population prevalence could be explained by the demographic profile of injury patients. In general, the trauma population tends to be younger and healthier than the general population. A number of prospective studies have shown pre-injury quality of life to be higher than age and gender matched population norms [35-37] and hence would be expected to have a lower prevalence of chronic diseases. However, the prevalence of some of these conditions (e.g. arthritis) is substantially lower than expected, particularly given the prevalence of elderly patients in the study sample and this is likely to reflect the ICD-10 coding practices and directives.

As noted in the methods, coders are directed to code conditions that have impacted on care provided during the patient’s hospital stay, and this will underestimate the true prevalence of chronic conditions. The finding that the models performed similarly is more likely to reflect the types of conditions coded from the medical record and the importance of these conditions to patient care. All indices included conditions such as diabetes and heart disease which require ongoing clinical management during a patient’s admission.

The overarching purpose of this study was to explore the relative contribution of each comorbidity measurement approach, and its contribution over age alone, to prediction of functional outcome. Importantly, despite models including age, injuries sustained and comorbidity, the capacity to discriminate between recovered/non-recovered patients was only in the acceptable range (AUC ≈ 0.70), confirming the importance of other factors. Numerous injury, personal and environmental factors have the capacity to influence the recovery of an individual. This is well demonstrated in the literature where factors such as compensable status [10,11,15,16,38,39], level of education [11,13,16], the intent of injury [11], gender [11,13,16,40,41], social circumstances [13,16,18], intent [11], mechanism of injury [11], and the level of designation of the trauma centre of management [11,42] have been shown to be important. While adjustment for comorbidity is important, the contribution of other factors cannot be under-estimated and should be part of any risk-adjustment processes.

The strengths of this study are the large number of cases (>13,000), high follow-up rate at 12-months (87% of all survivors to hospital discharge), and the use of a hospital discharge data to identify comorbidities which has been shown to have low error rates in diagnoses audited [43]. However, there were study limitations.

The study focused on orthopaedic trauma cases rather than all admitted trauma cases. While many cases had also sustained non-orthopaedic injuries, the results may not reflect cases without orthopaedic injury and this should be considered when interpreting the findings. Nevertheless, the orthopaedic registry was selected as the data source specifically for this study because this population includes a higher proportion of elderly patients with comorbidities than other injury populations such as major trauma. There were differences between included cases and those lost to follow-up at 12-months with a bias towards older patients with comorbidity in the group followed-up and included in this study. Secondly, there are challenges in collecting comorbidity information from patients from large populations and particularly where cognitive deficit (e.g. head injury and pre-existing dementia) are prevalent. Therefore, we were not able to assess the agreement between self-report and ICD-10 coding, or fully evaluate the relationship between the FCI and the six frequent chronic conditions not prevalent in the ICD-10 codes, highlighting issues with adapting self-report based indices (e.g. the six frequent chronic conditions described by Haagsma *et al.*) to ICD-10 based datasets. In future, the development of privacy protecting record linkage systems may enable primary care data to be linked to trauma and hospital discharge datasets to permit evaluation of the impact of conditions not deemed to influence in-hospital care.

Each of the participating hospitals’ coders used the same coding standard but the potential for variable interpretation of the medical record and the coding directives remains, despite regular auditing of the hospital discharge data. Additionally, ICD-10-AM coding is done in Australia for reimbursement purposes. In other jurisdictions, where ICD-10 coding is not used for reimbursement or where systems limit the number of codes recorded, the number and distribution of codes may differ. Whether this would impact on ICD-10 based comorbidity adjustment is unknown but warrants consideration in the interpretation of the findings and for further research. Finally, this study focused on functional outcome. The possibility that the relationship between comorbidity and other outcomes such as health-related quality of life, return to work and pain differs from the relationship between comorbidity and functional status will be examined in further research using data from VOTOR.

## Conclusions

Mapping of ICD-10 codes to comorbidity indices showed that comorbidity is an important predictor of long term functional outcome following orthopaedic trauma, independent of age and injuries sustained. Adjustment for comorbidity is indicated when assessing risk-adjusted functional outcomes over time or across jurisdictions.

## Competing interests

The authors declare that they have no competing interests.

## Authors’ contributions

All authors contributed to the conception and design and interpretation of data. BJG analysed the data for this study. BJG drafted the article and all authors reviewed it critically for important intellectual content. All authors approved the final version for submission.

## Pre-publication history

The pre-publication history for this paper can be accessed here:

http://www.biomedcentral.com/1472-6963/13/30/prepub

## References

[B1] Morris J Jr MacKenzie E Edelstein S The effect of preexisting conditions on mortality in trauma patients J Am Med Assoc 1990 263 14 1942 1946 10.1001/jama.1990.03440140068033 2313871

[B2] Tan C Ng A Civil I Co-morbidities in trauma patients: common and significant N Z Med J 2004 117 1201 1044 1049 15476004

[B3] Wardle T Co-morbid factors in trauma patients Brit Med Bull 1999 55 4 744 756 10.1258/0007142991902754 10746328

[B4] Bergeron E Rossignol M Osler T Clas D Lavoie A improving the TRISS methodology by restructuring age categories and adding comorbidities J Trauma 2004 56 4 760 767 10.1097/01.TA.0000119199.52226.C0 15187738

[B5] Gabbe B Magtengaard K Hannaford A Cameron P Is the Charlson Comorbidity Index useful for predicting trauma outcomes? Acad Emerg Med 2005 12 318 321 10.1111/j.1553-2712.2005.tb01950.x 15805322

[B6] McGwin G MacLennan P Bailey Fife J Davis G Rue L Preexisting conditions and mortality in older trauma patients J Trauma 2004 56 6 1291 1296 10.1097/01.TA.0000089354.02065.D0 15211139

[B7] Haagsma J van Beeck E Polinder S Toet H Panneman M Bonsel G The effect of comorbidity on health-related quality of life for injury patients in the first year following injury: comparison of three comorbidity adjustment approaches Pop Health Metr 2011 9 10 10.1186/1478-7954-9-10 PMC3096905 21513572

[B8] Polinder S Haagsma J Lyons R Gabbe B Ameratunga S Cryer C Derrett S Harrison J Segui-Gomez M van Beeck E Measuring the population burden of fatal and non-fatal injury Epidemiol Rev 2012 34 17 31 10.1093/epirev/mxr022 22113244

[B9] de Groot V Beckerman H Lankhorst G Bouter L How to measure comorbidity: a critical review of available methods J Clin Epidemiol 2003 56 3 221 229 10.1016/S0895-4356(02)00585-1 12725876

[B10] Cameron C Purdie D Kliewer E McClure R Differences in prevalence of pre-existing morbidity between injured and non-injured populations Bull World Health Org 2005 83 5 345 352 15976875 PMC2626228

[B11] Gabbe B Simpson P Sutherland A Wolfe R Fitzgerald M Judson R Cameron P Improved functional outcomes for major trauma patients in a regionalised, inclusive trauma system Ann Surg 2012 255 6 1009 1015 10.1097/SLA.0b013e31824c4b91 22584628

[B12] Haentjens P Autier P Barette M Venken K Vanderschueren D Boonen S Survival and functional outcome according to hip fracture type: A one-year prospective cohort study in elderly women with an intertrochanteric or femoral neck fracture Bone 2007 41 958 964 10.1016/j.bone.2007.08.026 17913614

[B13] Holtslag H Van Beeck E Lindeman E Leenen L Determinants of long-term functional consequences after major trauma J Trauma 2007 62 4 919 927 10.1097/01.ta.0000224124.47646.62 17426549

[B14] Kelley-Quon L Min L Morley E Hiatt J Cryer H Tillou A Functional status after injury: A longitudinal study of geriatric trauma Amer Surg 2010 76 10 1055 1058 21105608 PMC3144866

[B15] Polinder S Haagsma J Bonsel G Essink-Bot M Toet H van Beeck E The measurement of long-term health-related quality of life after injury: comparison of EQ-5D and the health utilities index Inj Prev 2010 16 147 153 10.1136/ip.2009.022418 20570982

[B16] Ringburg A Polinder S van Ierland M Steyerberg E van Lieshout E Patka P van Beeck E Schipper I Prevalence and prognostic factors of disability after major trauma J Trauma 2011 70 4 916 922 10.1097/TA.0b013e3181f6bce8 21045741

[B17] Edwards E Graves S McNeil J Williamson O Urquhart D Cicuttini F Orthopaedic trauma: Establishment of an outcomes registry to evaluate and monitor treatment effectiveness Injury 2006 37 95 96 10.1016/j.injury.2005.02.027 15979074

[B18] Gabbe B Sutherland A Hart M Cameron P Population-based capture of long-term functional and quality of life outcomes after major trauma - the experiences of the Victorian State Trauma Registry J Trauma 2010 69 3 532 536 10.1097/TA.0b013e3181e5125b 20838122

[B19] National Centre for Classification in Health International Statistical Classification of Diseases and Related Health Problems, Tenth Revision, Australian Modification (ICD-10-AM), the Australian Classification of Health Interventions (ACHI) and the Australian Coding Standards (ACS) 2010 7 Sydney

[B20] Groll D To T Bombardier C Wright J The development of a comorbidity index with physical function as the outcome J Clin Epidemiol 2005 58 595 602 10.1016/j.jclinepi.2004.10.018 15878473

[B21] Wilson J Pettigrew L Teasdale G Structured interviews for the Glasgow Outcome Scale and the Extended Glasgow Outcome Scale: Guidlines for their use J Neurotrauma 1998 15 8 573 585 10.1089/neu.1998.15.573 9726257

[B22] Gabbe B Williamson O Cameron P Dowrick A Choosing outcome assessment instruments for trauma registries Acad Emerg Med 2005 12 751 758 10.1111/j.1553-2712.2005.tb00943.x 16079429

[B23] Ardolino A Sleat G Willett K Outcome measurements in major trauma — Results of a consensus meeting Injury 2012 43 1662 1666 10.1016/j.injury.2012.05.008 22695320

[B24] Williamson O Gabbe B Forbes A Wolfe R Sutherland A Cameron P Comparing the responsiveness of functional outcome assessment instruments for trauma registries J Trauma 2011 71 1 63 68 10.1097/TA.0b013e31820e898d 21427612

[B25] Altman D Vergouwe Y Royston P Moons K Prognosis and prognostic research: validating a prognostic model Brit Med J 2009 338 b605 1432 1435 10.1136/bmj.b605 19477892

[B26] Hosmer D Lemeshow S Applied Logistic Regression, Second Edition edn 2000 New York: John Wiley & Sons, Inc.

[B27] Fan V Maciejewski M Liu C McDonell M Fihn S Comparison of risk adjustment measures based on self-report, administrative data, and pharmacy records to predict clinical outcomes Health Serv Outcomes Res Method 2006 6 21 36 10.1007/s10742-006-0004-1

[B28] Lash T Mor V Wieland D Ferrucci L Satariano W Silliman R Methodology, design, and analytic techniques to address measurement of comorbid disease J Gerontol A Biol Sci Med Sci 2007 62 3 281 285 10.1093/gerona/62.3.281 17389725 PMC2645650

[B29] Chong W Ding Y Heng B A comparison of comorbidities obtained from hospital administrative data and medical charts in older patients with pneumonia BMC Heal Serv Res 2011 11 105 10.1186/1472-6963-11-105 PMC3112394 21586172

[B30] March L Bagga H Epidemiology of osteoarthritis in Australia Med J Aust 2004 180 5 Suppl S6 S10 14984356 10.5694/j.1326-5377.2004.tb05906.x

[B31] Australian Institute of Health and Welfare A snapshot of arthritis in Australia 2010 Arthritis series no 13 2010 Canberra: Australian Institute of Health and Welfare http://www.aihw.gov.au/publication-detail/?id=6442468397&tab=2

[B32] Thorburn A Prevalence of obesity in Australia Obes Rev 2005 6 3 187 189 10.1111/j.1467-789X.2005.00187.x 16045631

[B33] Australian Institute of Health and Welfare Cardiovascular disease: Australian facts 2011 Cardiovascular disease series 2011 Canberra: Australian Institute of Health and Welfare http://www.aihw.gov.au/publication-detail/?id=10737418510&tab=2

[B34] Australian Institute of Health and Welfare Diabetes prevalence in Australia Diabetes Series 2009 Canberra: Australian Institute of Health and Welfare http://www.aihw.gov.au/publication-detail/?id=6442468288&tab=2

[B35] Gabbe B Cameron P Graves S Edwards E Pre-injury status: Are orthopaedic trauma patients different to the general population? J Orthop Trauma 2007 21 4 223 228 10.1097/BOT.0b013e31803eb13c 17414548

[B36] Lyons R Kendrick D Towner E Christie N Macey S Gabbe B Measuring the population burden of injuries - Implications for global and national estimates: A multi-centre prospective UK longitudinal study PLoS Med 2011 8 12 e1001140 10.1371/journal.pmed.1001140 22162954 PMC3232198

[B37] Watson W Ozanne-Smith J Richardson J Retrospective baseline measurement of self-reported health status and health-related quality of life versus population norms in the evaluatin of post-injury losses Inj Prev 2007 13 45 50 10.1136/ip.2005.010157 17296689 PMC2610562

[B38] Gabbe B Cameron P Williamson O Edwards E Graves S Richardson M The relationship between compensable status and long-term patient outcomes following orthopaedic trauma Med J Aust 2007 187 1 14 17 17605697 10.5694/j.1326-5377.2007.tb01108.x

[B39] Harris I Young J Rae H Jalaludin B Solomon M Predictors of general health after major trauma J Trauma 2008 64 4 969 974 10.1097/01.ta.0000245972.83948.1a 18404063

[B40] Holbrook T Hoyt D Anderson J The importance of gender on outcomes after major trauma: functional and psychologic outcomes in women vesus men J Trauma 2001 50 270 273 10.1097/00005373-200102000-00012 11242291

[B41] Vles W Steyerberg E Essink-Bot M van Beeck E Meeuwis J Leenen L Prevalence and determinants of disabilities and return to work after major trauma J Trauma 2005 58 126 135 10.1097/01.TA.0000112342.40296.1F 15674163

[B42] MacKenzie E Rivara F Jurkovich G Nathens A Egleston B Salkever D Frey K Scharfstein D The impact of trauma-center care on functional outcomes following major lower limb trauma J Bone Joint Surg 2008 90-A 1 101 109 18171963 10.2106/JBJS.F.01225

[B43] Henderson T Shepheard J Sundararajan V Quality of diagnosis and procedure coding in ICD-10 administrative data Med Care 2006 44 11 1011 1019 10.1097/01.mlr.0000228018.48783.34 17063133

